# Complete mitochondrial genome pathological characteristics and scanning electron microscopic observations of *Armillifer moniliformis* isolated from *Manis javanica*

**DOI:** 10.1016/j.ijppaw.2025.101183

**Published:** 2026-01-03

**Authors:** Xianghe Wang, Sitong Chen, Fuyu An, Xinyu Liu, Hongmei Yan, Zhenquan Zhang, Zi-Guo Yuan, Yan Hua

**Affiliations:** a Guangdong Provincial Key Laboratory of Silviculture, Protection and Utilization, Guangdong Academy of Forestry, Guangzhou, 510520, China; b Guangdong Provincial Key Laboratory of Zoonosis Prevention and Control, College of Veterinary Medicine, South China Agricultural University, Guangzhou, China

**Keywords:** *Armillifer moniliformis*, *Manis javanica*, Mitochondrial genome, Scanning electron microscopy

## Abstract

*Armillifer moniliformis* is a multi-host parasite, with previous studies documenting various wild animals as well as humans serving as its intermediate hosts. However, to date, there have been no reports of pangolins being infected with this parasite. Necropsy revealed *Armillifer moniliformis* formed cysts on the abdominal greater omentum and multiple organs (lungs, liver, etc.). Scanning electron microscopy showed its typical structures (oral hooks, abdominal annuli), while histopathological examination indicated infected organs exhibited congestion, inflammatory infiltration, and calcification. At the molecular level, identification based on the 18S rRNA sequence demonstrated that this parasite shares 99.89 % homology with *Armillifer moniliformis* and has a close phylogenetic relationship with it. In addition, *Armillifer moniliformis* parasites were isolated from the host, and their DNA was extracted for Illumina and HiFi sequencing, resulting in the assembly of the complete mitochondrial genome of *Armillifer moniliformis.* The assembled circular mitochondrial genome had a total length of 16516 bp and comprised 13 unique protein-coding genes, 22 tRNA genes, and 2 rRNA genes. The overall nucleotide composition of *Armillifer moniliformis* was 62 % A + T and 38 % G + C. The protein-coding genes of the parasite encode a total of 3538 amino acids, utilizing four types of initiation codons (ATA, ATC, ATT, and ATG) and three types of termination codons (TAA, TAG, and T). The genome contained 4 simple sequence repeats (SSRs) and 158 dispersed repeats (≥20 bp). Phylogenetic trees based on 13 protein-coding genes showed *Armillifer* and *Linguatula* formed a Pentastomida clade (100 % bootstrap support), which together with Branchiura and Ostracoda constitutes Oligostraca; Crustacea and Hexapoda form a monophyletic Pancrustacea clade. In this study, an *Armillifer moniliformis* parasite was isolated and identified from the Malayan pangolin. This work not only expands our understanding of *Armillifer moniliformis* species but also provides a crucial foundation for further investigations into their taxonomy, diagnostics, and biological characteristics.

## Highlights

• First report of *Armillifer moniliformis* infection in Malayan pangolins, expanding its known host range.
• SEM shows oral hooks and abdominal annuli; pathology shows congestion, inflammation and calcification in lung and liver.
• 18S rRNA confirms *A. moniliformis*; the complete mitogenome (16,516 bp) was assembled and annotated.
• Phylogeny using 13 PCGs supports monophyletic Pentastomida and places it within Oligostraca.

## Graphical abstract

**Figure:**
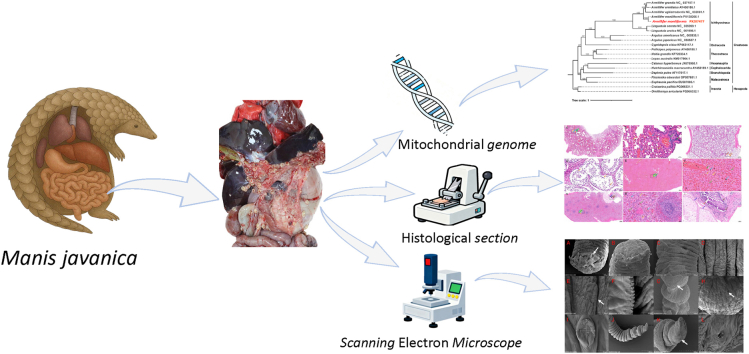

## 1 Introduction

*Armillifer* sp. Belongs to the family Porocephalidae. To date, eight species have been recognized within this genus: *Armillifer aborealis, Armillifer agkistrodontis, Armillifer armillatus, Armillifer australis, Armillifer grandis, Armillifer mazzai, Armillifer moniliformis,* and *Armillifer yoshidai.* This parasite has a worldwide distribution, with adult parasites primarily parasitizing the respiratory tracts of reptiles, particularly snakes. The nymphal stages of this parasite exhibit a broad host range, capable of infecting humans as well as other wild animals. They primarily inhabit the surfaces of internal organs, and transmission occurs mainly through infectious eggs excreted in the feces and oral or nasal secretions of the definitive hosts. It was first described in human disease in 1847 and can cause pentastomiasis in humans(Christoffersen and De Assis, 2013; Hardi et al., 2017; Rajapaksha et al., 2020; Chen et al., 2010; Junker and de Klerk-Lorist, 2020; Li et al., 2024, Li et al., 2024). *Armillifer* sp. Infections are primarily transmitted through oral ingestion, such as drinking water contaminated with parasite eggs or consuming snake blood. Once the infectious eggs enter an intermediate host, they develop into nymphs and migrate to the surfaces of internal organs, where they form cysts. In severe cases, infection can result in death in both humans and animals(Lavarde and Fornes, 1999; Slocombe and Budd, 1973).

There are eight extant species of pangolins worldwide. In China, two species are found: the Chinese pangolin (*Manis pentadactyla*), mainly distributed in Jiangxi, Fujian, Zhejiang, Guangdong, Anhui, and Hainan provinces, as well as Taiwan, and the Sunda pangolin (*Manis javanica*), mainly distributed in Yunnan Province(Yan et al., 2021; Zhang et al., 2021). *Manis javanica* primarily feeds on ants and ingests substantial amounts of soil during foraging. Notably, both ants and soil often harbor large numbers of parasite eggs, and this dual exposure significantly increases the likelihood of parasitic infection in this species(Yan et al., 2021; Belden and Harris, 2007). Previous studies have reported that pangolins, including *Manis pentadactyla* and *Manis javanica*, can be infected with a variety of parasites, such as *Ancylostoma* sp., *Raillietina* tapeworms, *Spiruromorpha* sp., Acanthocephala, and *Eimeria* sp(Barton et al., 2022; Mohapatra et al., 2016; Tuli et al., 2022a, 2022b; Li et al., 2024; Vibulchan et al., 2024). However, mitochondrial genome data for pangolin parasites are currently limited to *Ancylostoma* sp. and *Raillietina* tapeworms. To date, there have been no reported cases of *A. moniliformis* infection causing death in *Manis pentadactyla* or *Manis javanica*.

This study reports the death of an adult male *Manis javanica* caused by *A*. *moniliformis* infection. The parasites were first examined using Scanning Electron Microscopy (SEM) to characterize their morphological features in detail, and histopathological analyses were performed on the tissues of the deceased animal. The study systematically characterized the lesions induced by *A. moniliformis* infection across multiple tissues and organs. Subsequently, DNA was extracted from the parasites and subjected to Illumina and HiFi sequencing, resulting in the successful assembly of the complete mitochondrial genome of *A. moniliformis.*

## 2 Methods

### 2.1 Parasite collection

A consignment of *Manis javanica* suspected of being illegally trafficked was intercepted at customs. Among them, a male individual, subjected to prolonged transportation, traumatic injuries, and stress, received intensive veterinary care at the Guangdong Wildlife Monitoring and Rescue Center. Despite these efforts, the animal ultimately succumbed to its injuries. Upon necropsy of the deceased animal, nearly one hundred parasites were observed on the surfaces of various abdominal organs. The parasites were rinsed with Phosphate Buffered Saline (PBS) and then preserved in 75 % ethanol, stored at −20 °C for subsequent analysis.

### 2.2 Scanning Electron Microscopy sample preparation

The serosal layers of the deceased animal's organs were carefully incised to retrieve fresh parasites. The parasites were gently rinsed with PBS and then fixed in electron microscopy fixative at room temperature for 2 h, followed by storage at 4 °C. The fixed samples were rinsed three times with 0.1 M phosphate buffer (pH 7.4), with each rinse lasting 15 min. The samples were post-fixed in 1 % osmium tetroxide, prepared in 0.1 M phosphate buffer (pH 7.4), at room temperature, protected from light, for 1–2 h. The samples were rinsed three times with 0.1 M phosphate buffer (pH 7.4), with each rinse lasting 15 min. The tissues were then dehydrated sequentially in an ethanol series at concentrations of 30 %, 50 %, 70 %, 80 %, 90 %, 95 %, and twice in 100 %, with each step lasting 15 min, followed by a 15 min treatment in isoamyl acetate. The samples were dried using a critical-point dryer, then mounted onto a conductive carbon tape on an SEM stub and sputter-coated with gold for 30 s.

### 2.3 Histological sample processing

During necropsy of the deceased animal, numerous *A. moniliformis* nymphs were observed in the omental tissue of the abdominal cavity. The affected tissues were fixed in 4 % paraformaldehyde (at a volume ten times that of the specimen) for 36 h, followed by graded ethanol dehydration (70 %, 80 %, 90 %, 95 %, and 100 %), xylene clearing, and paraffin infiltration. The samples were subsequently embedded in paraffin to obtain tissue blocks. After sectioning with a microtome, tissue ribbons were floated on a 40–45 °C water bath to flatten, and sections were subsequently mounted onto glass slides. The slides were baked in a 60–65 °C oven for 2–4 h to ensure firm adhesion of the sections. Deparaffinization was carried out in two successive xylene baths (10 min each), followed by rehydration through a graded ethanol series: absolute ethanol I and II (5 min each), 95 %, 90 %, 80 %, and 70 % ethanol (3–5 min each). Finally, the sections were rinsed with distilled water 2–3 times (1–2 min each). Sections were immersed in hematoxylin solution for 10 min to stain the nuclei, followed by running tap water for 10–15 min to remove excess dye and promote color development. When overstaining occurred, differentiation was performed in 1 % acid alcohol for approximately 10 s, until the nuclei were clearly visible under the microscope and the cytoplasm appeared nearly colorless. Differentiation was immediately terminated by rinsing in running tap water, and washing was continued for an additional 15 min until the sections turned blue. Subsequently, sections were counterstained in eosin solution for 1–5 min to visualize the cytoplasm and extracellular matrix, and then briefly rinsed with distilled water to remove excess dye. Sections were dehydrated in 95 % ethanol I and II (1 min each), followed by absolute ethanol I and II (2 min each), and cleared in xylene I and II (3 min each). A drop of neutral balsam was applied to the center of each section, and a coverslip was carefully mounted while avoiding air bubbles. The slides were then air-dried under ventilation or baked at 60 °C for 30 min to complete mounting. After the mounting medium had dried, tissue morphology was examined under a light microscope.

### 2.4 DNA extraction and whole genome amplification

gDNA samples for short-read sequencing were extracted from tender leaves using the CTAB method. Library preparation and sequencing (DNBSEQ-T7RS) Libraries were constructed by MGlEasy Universal DNA Library Prep Kit V1.0 (CAT#1000005250, MGl) following thestandard protocol. Briefly, 1 μg genomic DNA was randomly fragmented by Covaris. The fragmented DNA was selected by MGlEasy DNA Clean beads (CAT#1000005279, MGl) to an average size of 200–400 bp. After that, the selected fragments were end-repaired, 3′ adenylated and then ligated adapters. The DNAsamples were amplified by PCR and the products were purified by the MGlEasyDNA Clean beads (CAT#1000005279, MGl). The double stranded PCR products were heat denatured and circularized by the splint oligo sequence in MGlEasy Circularization Module (CAT#1000005260, MGl). The single strand circle DNA (ssCirDNA) were formatted as the final library and qualified by QC. The qualified libraries were sequenced on DNBSEQ-T7RS platform.

The amplification mixture, containing 10X Amplification Master Mix, WGA DNA Polymerase, and nuclease-free water, was subjected to PCR with the following conditions: initial denaturation at 95 °C for 3 min; 14 cycles of denaturation at 95 °C for 15 s and annealing/extension at 65 °C for 5 min. The WGA product was purified using the GenElute PCR Purification Kit (NA1020, Sigma-Aldrich) according to the manufacturer's instructions. Sequencing libraries were then constructed from the amplified DNA using the VAHTS Universal Plus DNA Library Prep Kit for Illumina V2 (Vazyme, Nanjing, PRC). The procedure included fragmentation (though note the WGA product was already fragmented), end repair, 'A'-tailing, adapter ligation, DNA purification, and PCR amplification. The resulting Illumina-compatible libraries were quantified using a Qubit 4.0 Fluorometer (Life Technologies, Carlsbad, CA, USA) and diluted to a working concentration of 1 ng/μL. The fragment size distribution of the final libraries was assessed using an Agilent 2100 Bioanalyzer (Agilent Technologies, Santa Clara, CA, USA). Quantitative PCR was further employed for accurate concentration measurement to ensure optimal sequencing cluster generation. The libraries were sequenced on an Illumina NovaSeq X Plus (Illumina Inc., CA, USA) platform to generate 150-bp paired-end reads.

### 2.5 Protocol of PacBio HiFi library construction and sequencing

The PacBio HiFi library was constructed to generate long-read, high-fidelity data. Briefly, 7 μg of high-quality genomic DNA (pre-WGA) was sheared to a target fragment size of approximately 15–20 kb using the Megaruptor 3 system (Diagenode) according to the manufacturer's recommendations. The sheared DNA was purified using 1X volume of magnetic beads (e.g., AMPure PB beads) and eluted in 47 μL of low TE buffer. The quantity and quality of the sheared DNA were assessed using a Qubit Fluorometer. The SMRTbell library was constructed using the SMRTbell Prep Kit 3.0 (Pacific Biosciences). The process involved DNA damage repair, end repair, and ligation of hairpin adapters to both ends of the DNA fragments, creating a circularized template suitable for PacBio sequencing. The ligated product was purified to remove excess adapters and short fragments. Enzymatic treatment with a nuclease mix (e.g., a combination of Exonuclease III and Exonuclease VII) was performed to digest any unligated linear DNA molecules and damaged SMRTbell templates, enriching for intact, circularized libraries. The final library was size-selected using the BluePippin or Sage ELF System (e.g., for a 15–20 kb cutoff) to ensure insert size homogeneity. The prepared SMRTbell library was annealed to sequencing primers and bound to the proprietary DNA polymerase according to the standard Pacific Biosciences binding protocol. Sequencing was performed on the PacBio Sequel II system (or Revio system, if applicable) using the Circular Consensus Sequencing (CCS) mode. In CCS mode, each circular DNA template is sequenced multiple times as the polymerase traverses it repeatedly. The resulting raw data, comprising multiple subreads for each template, was processed using the CCS2 (SMRT Link) software (minimum number of passes = 3, minimum predicted accuracy >0.99) to generate highly accurate HiFi reads (read length 10–20 kb, accuracy >99.9 %).

### 2.6 Data quality control and public data deposition

Prior to assembly, raw sequencing data from all platforms (Illumina, PacBio) underwent rigorous quality control. For Illumina short reads, quality control was performed using fastp (version 0.23.2) with default parameters to automatically remove adapter sequences, trim low-quality bases (quality threshold <20), and discard reads with a length below 50 bp or containing ambiguous bases ('N') exceeding 5 %. For PacBio HiFi reads, the quality was inherently high due to the CCS process; however, basic filtering based on length and quality scores was confirmed during the CCS step. The quality of the final HiFi reads was assessed using standard PacBio analysis tools within SMRT Link.

### 2.7 PCR amplification and DNA sequencing

Taq DNA polymerase was used for amplification with the primers 18S-F:AACCTGGTTGATCCTGCCAGT, 18S-R:GATCCTTCTGCAGGTTCACCTAC(Li et al., 2024). DNA was extracted from the parasites and used to amplify the 18S rRNA gene of *A. moniliformis* PCR reactions were conducted using Taq PCR Mastermix (Vazyme, Nanjing, China) with a total reaction volume of 25 μL. The components included: 1 μL of forward primer, 1 μL of reverse primer, 12.5 μL of Taq PCR Mastermix, 8.5 μL of enzyme-free ddH_2_O, and 2 μL of DNA. The PCR conditions were set as follows: initial denaturation at 94 °C for 5 min, followed by 35 cycles of 94 °C for 20 s, 54 °C for 20 s, and 72 °C for 2 min and 10 s. After cycling, a final extension was performed at 72 °C for 10 min, and the products were subsequently stored at 4 °C until further use. Agarose Gel DNA Recovery Kit (TIANGEN, DP219-02) was used for gel extraction. The recovered product was subjected to TA cloning using the pMD 19-T Vector (Takara, 6013). The cloned product was introduced into competent *Escherichia coli* DH5α cells via heat shock transformation, followed by incubation at 37 °C for 12 h on Luria–Bertani (LB) agar plates containing ampicillin, IPTG, and X-Gal for blue-white colony selection. White colonies from each plate were picked, and positive insert fragments were verified by colony PCR. Six positive colonies were inoculated into LB liquid medium supplemented with ampicillin and cultured for 6 h. Plasmids were extracted using the Plasmid Mini-Prep Kit (TIANGEN, DP103). Sanger sequencing was performed by Sangon Biotech (Shanghai) Co., Ltd. Using M13–47 and M13-48 universal primers.

### 2.8 Complete mitochondrial genome of the parasite

A total of 15 GB of raw data was obtained using both Illumina sequencing and HiFi sequencing. The mitochondrial genome was assembled using a hybrid assembly strategy combining second-generation sequencing data from Illumina/BGI and third-generation sequencing data from Pacbio HiFi and illumina. First, the second-generation DNA sequencing data were used for reference-free mitochondrial genome assembly with MitoZ software, generating a long scaffold. Subsequently, the MitoZ assembly was used as a reference, and the Illumina short-read DNA sequencing data were subjected to reference-guided mitochondrial assembly with GetOrganelle, yielding long scaffolds. Subsequently, the assemblies generated from Illumina short-read data were used as reference sequences to filter the raw long-read data with Minimap2. The filtered long-read DNA sequencing data were then subjected to mitochondrial genome assembly using Flye, resulting in a circularized long-read assembly. Assembly. The assembly generated with GetOrganelle from Illumina short-read data was oriented consistently with the Flye long-read assembly, and the gaps present in the short-read assembly were bridged using the long-read data, yielding a circularized mitochondrial genome. The resulting mitochondrial genome of *A*. *moniliformis* was subsequently visualized with OGDRAW.

### 2.9 Gene annotation and sequence analysis

Annotation of the complete mitochondrial genome was performed with MitoZ and MITOS. The appropriate reference database was selected according to the species information of the mitochondrial genome. Additionally, a total of 13 unique protein-coding genes, 22 tRNA genes, and 2 rRNA genes were annotated. The protein-coding genes include two ATP synthase genes (*atp6* and *atp8*) and seven NADH dehydrogenase genes (*nad1*, *nad2*, *nad3*, *nad*4, *naad4L*, *nad5*, and *nad6*). Three cytochrome *c* oxidase genes (*cox1*, *cox2*, and *cox3*) and one cytochrome *b* gene (*cytb*) were also identified. Protein-coding sequences of the genome were extracted using PhyloSuite. Codon usage bias of the mitochondrial protein-coding genes was analyzed using MEGA 7.0, and the relative synonymous codon usage (RSCU) values were calculated. Repetitive sequences, including microsatellites, tandem repeats, and dispersed repeats, were identified using MISA (https://webblast.ipk-gatersleben.de/misa/)、TRF (V4.09) (https://tandem.bu.edu/trf/trf.unix.help.htm1), and the REPuter web server (https://bibiserv.cebitec.uni-bielefeld.de/reputer/). The results were visualized using Microsoft Excel and the Circos package (v0.69-9).

### 2.10 Phylogenetic analysis of parasite genomes based on 18S rRNA and protein-coding genes (PCGs)

We obtained 18S rRNA sequences of 6 parasites from the NCBI database and used these, along with the amplified 18S rRNA of the parasites, to construct a phylogenetic tree (Additional file 1: Table S1). The maximum likelihood (ML) method was employed to evaluate the phylogenetic tree, and the ML tree was constructed using the K2 + I model in MEGA12. A bootstrap value of greater than 70 % was considered strong support.

Based on phylogenetic relationships, closely related species were selected, and their mitochondrial genomes were downloaded (according to the species included in this study). Shared genes were then extracted using PhyloSuite (v1.1.16)(Zhang et al., 2020). Multiple sequence alignments were performed using MAFFT (v7.505)(Katoh and Standley, 2013). Phylogenetic trees were then constructed using IQ-TREE (v1.6.12) based on the maximum likelihood (ML) method, with the parameters set as ‘--alrt 1000 -B 1000’(Nguyen et al., 2015). The results of the phylogenetic analysis were visualized using iTOL (v6). Phylogenetic trees were constructed based on the maximum likelihood (ML) method, with parameters set as ‘--alrt 1000 -B 1000’. The results of the phylogenetic analysis were visualized using iTOL (v6).

## 3 Results

### 3.1 Morphological observations and histopathological analysis of parasites

**Fig. 1:**
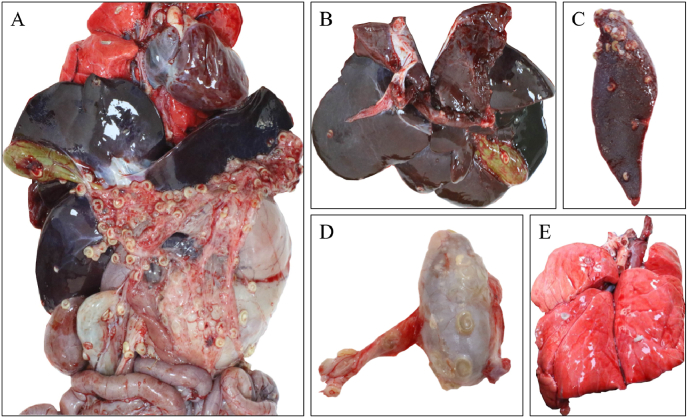
Necropsy Findings of a Deceased Male Malayan Pangolin A Numerous parasites attached to the greater omentum; Fig. B Parasites present on the liver and gallbladder; Fig. C Parasites on the surface of the spleen; Fig. D Parasites on the testes and tunica vaginalis of the spermatic cord; Fig. E Parasites on the surface of the lungs.

Necropsy of the deceased animals revealed numerous *A. moniliformis* nymphs on the greater omentum of the abdominal cavity, forming C-shaped translucent cysts and calcified cysts. A heavy parasite burden was also observed on the surfaces of the lungs, liver, spleen, gallbladder, testes, and stomach. The parasites collected during necropsy were cylindrical and pale yellow in color (Fig. 1). After collection, the specimens were rinsed with PBS and preserved in 70 % ethanol for subsequent experiments.

### 3.2 Scanning Electron Microscopy

**Fig. 2:**
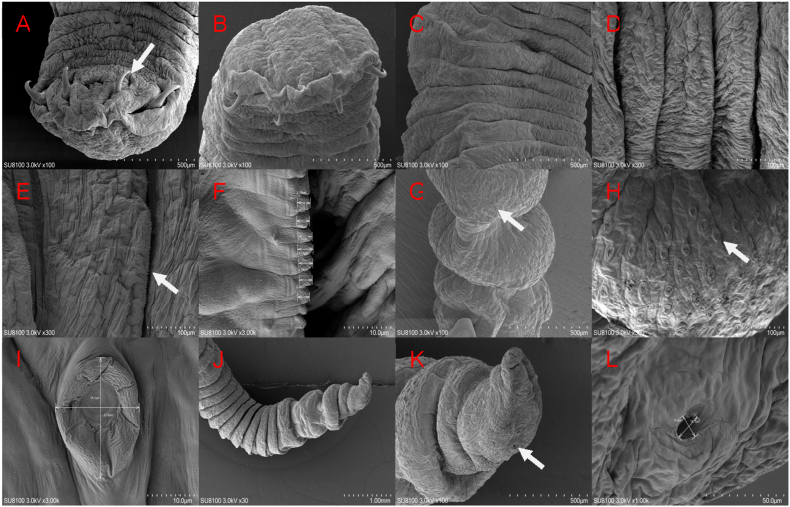
Scanning Electron Micrograph of *Armillifer moniliformis*. A BThe anterior end of *Armillifer moniliformis* has 4 prominent oral hooks (white arrow), and its body surface possesses waved folds; C D E F. The nymph's abdominal annuli are relatively wide, and on their undersides, there are neatly arranged serrated protrusions with a size of approximately 2.55–3.34 μm (white arrow); G H I. Sensory papillae on the incomplete annulus and measures approximately 22.9 μm × 18.1 μm (white arrow); J K L. The excretory pore opening at the nymph's posterior end is located behind the last incomplete annulus. It is circular in shape and measures approximately 16.4 μm × 14.1 μm (white arrow).

Scanning electron microscopy revealed that the parasite consists primarily of oral hooks, a mouth apparatus, abdominal annuli, incomplete annuli, and an excretory pore. The parasite's mouth is flattened, with four oral hooks located beneath it, arranged symmetrically on both sides of the oral opening. Its abdominal annuli extend from the anterior to the posterior end, with the middle abdominal annuli being the widest these are covered with irregularly arranged serrated protrusions and the posterior abdominal annuli the narrowest. Incomplete annuli near the posterior end have a large number of sensory papillae, measuring approximately 22.9 μm × 18.1 μm. Furthermore, an excretory pore is visible at the parasite's tail end, with a size of approximately 14.1 μm × 16.4 μm (Fig. 2).

### 3.3 Pathological findings

**Fig. 3:**
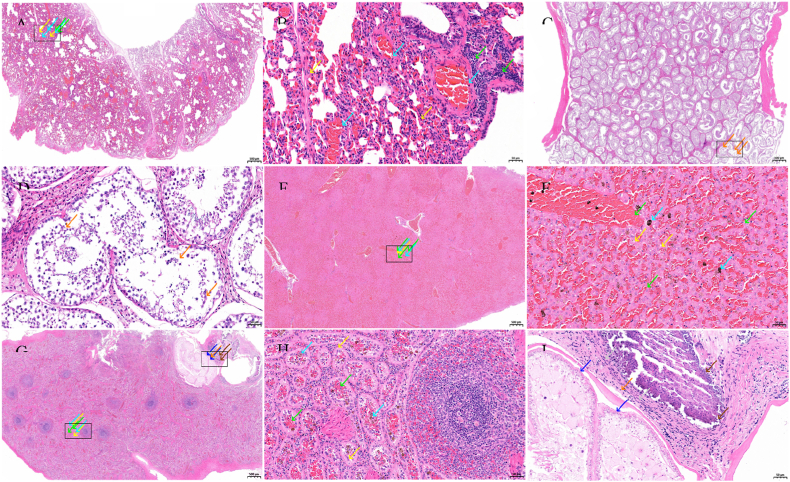
Histopathological Sections of the Lungs, Testes, Liver, and Spleen from a Deceased *Manis javanica* A and B: Lung tissues showing granulocytic infiltration within the alveolar walls (yellow arrows), widespread mild thickening of the alveolar septa, narrowing of multiple alveolar spaces with variable alveolar volumes, and focal perivascular and peribronchiolar lymphocytic infiltration (green arrows). Marked congestion is also observed (cyan arrows). The black boxes indicate the regions of magnified views. C and D: Testicular tissues showing extensive sloughing of spermatogenic epithelial cells from the seminiferous tubules (orange arrows). A few tubules contain sparse, loosely arranged, and irregularly organized cells, with a reduced number of spermatozoa in the lumen. The black boxes indicate the regions of magnified views. Fig. E and F: Hepatic tissues showing widespread congestion in the veins and hepatic sinusoids (green arrows), extensive sinusoidal dilation, and widening of the hepatic cords. A few hepatocytes exhibit swelling, necrotic degeneration, and contain small, round cytoplasmic vacuoles (yellow arrows). Scattered brownish-yellow pigment deposition is also observed (cyan arrows). No obvious necrosis or other notable abnormalities are detected. The black boxes indicate the regions of magnified views. Fig. G, H, and I: Splenic tissues showing indistinct boundaries between the red and white pulp. Widespread sinusoidal epithelial cells are arranged in a single layer of cuboidal cells (yellow arrows), with large cell bodies, orderly arrangement, and round or oval nuclei, accompanied by sinusoidal dilation and congestion (green arrows) with enlarged luminal spaces. Scattered brownish-yellow pigment deposition is also observed (cyan arrows). In regions with unclear structure, parasite-induced cavities lined by a single layer of epithelium are present (blue arrows), displaying irregular shapes, with surrounding calcified foci (brown arrows) encircled by sparse connective tissue proliferation (gray arrows), and occasional lymphocytes (orange arrows). The black boxes indicate the regions of magnified views.

Histopathological examination revealed vascular congestion in the alveolar septa, a small number of macrophages at the alveolar margins, and infiltration of granulocytes. Some alveoli contained serous exudates, and areas of calcification were observed. In the spleen, parasites and granulomas were observed, along with epithelioid cells, foreign body macrophages, numerous eosinophils, and lymphocytes. The boundary between the red and white pulp was indistinct, the splenic cords were loose and degenerated, and hemosiderin deposits were scattered throughout. Liver (congested): Hepatic cords contained abundant erythrocytes, and numerous inflammatory cells were observed in the portal areas. Hepatocytes exhibited swelling and necrosis with hydropic degeneration, and some hepatocytes showed fatty degeneration with lipid droplet accumulation. The surface tunica of the testicular tissue was composed of irregular dense connective tissue. Shedding of spermatogenic epithelial cells from the seminiferous tubules was observed, whereas no obvious necrosis or inflammatory cell infiltration was detected (Fig. 3).

### 3.4 Primary identification of parasites by molecular markers

**Fig. 4:**
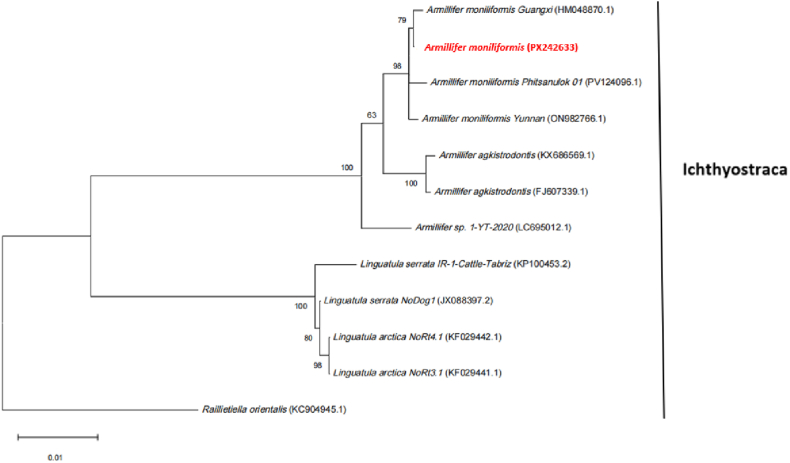
Phylogenetic tree of 18S rDNA sequences from *Armillifer moniliformis*. Phylogenetic trees were constructed using MEGA12 and the maximum likelihood method (ML method). A Bootstrap test was performed with 1000 replicates, and branches with Bootstrap values greater than 70 are considered highly reliable.

The 18S rRNA sequence of the parasite was amplified, yielding a fragment of 1834 bp (GenBank database under accession numbers: PX242633). Sequence analysis revealed 99.89 % homology with the 18S rRNA sequence of *A*. *moniliformis* in GenBank (GenBank database under accession numbers: ON982766.1) (Fig. 4). The phylogenetic tree based on the 18S rDNA sequence showed that the parasite (*Armillifer moniliformis*, PX242633) clustered together with other *A. moniliformis* sequences, *A. agkistrodontis*, and other *Armillifer* species, with the closest phylogenetic relationship to *A. moniliformis.* The selection process and results of the best evolutionary model for tree-building have been added to Supplementary Fig. S2.

### 3.5 Mitochondrial genome of the parasite

**Fig. 5:**
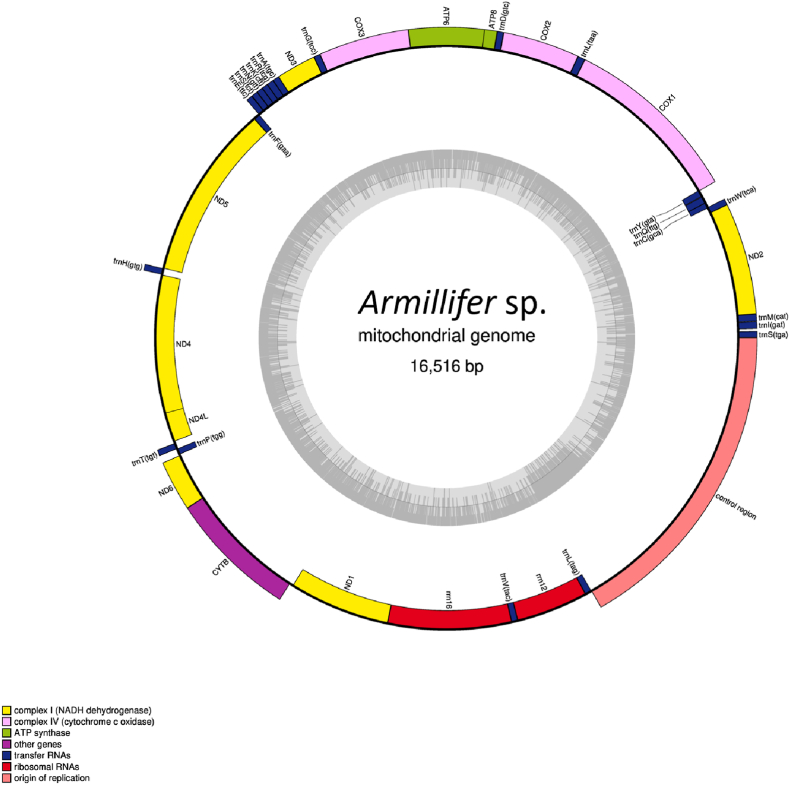
Mitochondrial genome map showing 13 protein-coding genes (PCGs), 22 tRNA genes, and 2 rRNA genes. The protein-coding genes include two ATP synthase genes (*atp6* and *atp8*), seven NADH dehydrogenase genes (*nad1*, *nad2*, *nad3*, *nad4*, *nad4L*, *nad5*, and *nad6*), three cytochrome *c* oxidase genes (*cox1*, *cox2*, and *cox3*), and one cytochrome *b* gene (*cytb*). The transcriptional orientations of the 13 PCGs are not uniform.

The mitochondrial genome of *A. moniliformis* is composed of a single circular scaffold with a total length of 16516 bp (GenBank database under accession number: PX207477). The annotated map is shown in Fig. 5, and the genes encoded by the mitochondrial genome are listed in Table 1. The nucleotide composition of the parasite's mitochondrial genome was A = 5,571, T = 4,670, C = 5,039, and G = 1,236, with an overall AT content of 62 % and CG content of 38 %. The AT/CG ratio of the mitochondrial genome was 1.63. Fourteen genes, including four PCGs (*nad5*, *nad4*, *nad4L*, and *nad1*) and eight tRNA genes (*trnC*, *trnG*, *trnT*, *trnP*, *trnH*, *trnP*, *trnV*, and *trnL*), as well as two rRNAs (*rrn16* and *rrn12*), were encoded on the H-strand. The remaining 23 genes were encoded on the L-strand (Table 2). Additionally, a long NCR spanning 2765 bp was identified between trnL and *trnS* (Supplementary Table S5).

**Table 1:** Genes encoded by the mitochondrial genome.

Group of genes	Name of genes
ATP synthase	*atp6、atp*8
NADH dehydrogenase	*nad*1*、nad*2*、nad*3*、nad*4*、nad*4L*、nad*5*、nad*6
Cytochrome *c* oxidase	*cox*1*、cox*2*、cox*3
Cytochrome *c* biogenesis	*cytb*
Ribosome RNA	*rrn*12S,*rrn*16S
Transfer RNA	*trnA, trnC, trnD, trnE, trnF, trnG, trnH, trnI, trnK, trnL1, trnL2, trnM, trnN, trnP, trnQ, trnR, trnS1, trnS2, trnT, trnV, trnW, trnY,*

**Table 2:** Molecular detection of a novel *Armillifer moniliformis* by whole mtDNA sequence from pangolin *Manis javanica*.

Gene/region	Position	Length		Codon		Anticodon
	Start to end	Number of nucleotides	Number of amino acids	Start	Stop	
*trnS*	1–55	55				TGA
*trnI*	76–137	62				GAT
*trnM*	140–201	62				CAT
*nad2*	202–1164	963	320	ATT	TAG	
*trnW*	1163–1218	56				TCA
*trnC*	1263–1211	53				GCA
*trnQ*	1327–1262	66				TTG
*trnY*	1390–1330	61				GTA
*cox1*	1383–2916	1534	511	ATC	T	
*trnL*	2917–2979	63				TAA
*cox2*	2980–3643	664	221	ATA	T	
*trnD*	3644–3698	55				GTC
*atp8*	3699–3809	111	36	ATT	TAA	
*atp6*	3806–4456	651	216	ATA	TAA	
*cox3*	4456–5236	781	260	ATG	T	
*trnG*	5237–5291	55				TCC
*nad3*	5292–5636	345	114	ATC	TAA	
*trnA*	5635–5691	57				TGC
*trnR*	5691–5751	61				TCG
*trnK*	5751–5804	54				CTT
*trnN*	5804–5858	55				GTT
*trnS*	5859–5913	55				TCT
*trnE*	5915–5969	55				TTC
*trnF*	6024–5968	57				GAA
*nad5*	7627–6025	1603	534	ATG	T	
*trnH*	7629–7684	56				GTG
*nad4*	8950–7687	1264	421	ATG	T	
*nad4L*	9213–8944	270	89	ATG	TAG	
*trnT*	9230–9284	55				TGT
*trnP*	9340–9285	56				TGG
*nad6*	9348–9785	438	145	ATA	TAA	
*cytb*	9785–10894	1110	369	ATG	TAA	
*nad1*	11848–10925	909	302	ATG	TAA	
*rrn16*	12977–11849	1129				
*trnV*	13034–12978	57				TAC
*rrn12*	13691–13035	639				
*trnL*	13751–13692	60				TAG
*NCR*	13752–16516	2765				

### 3.6 Codon usage bias analysis

The 13 PCGs had a total length of 10643 base pairs (bp), accounting for 64.4 % of the entire mitochondrial genome. The lengths of these PCGs ranged from the shortest, 111 bp (*atp8*), to the longest, 1603 bp (*nad5*). The overall nucleotide composition of the PCGs in the parasite's mitochondrial genome was A = 2833 (26 %), T = 3592 (38 %), C = 2579 (24 %), and G = 1639 (12 %). The AT skew [(A-T)/(A + T)] was −0.11 and the GC skew [(G-C)/(G + C)] was −0.22, indicating a pronounced AT bias, with thymine being the most abundant nucleotide and guanine the least abundan. The AT and GC skew values for all 13 PCGs are presented in Additional file 1: Table S3.

The mitochondrial PCG set of the parasite comprises 3538 amino acids and utilizes four distinct types of start codons (ATA, ATC, ATT, and ATG) as well as three types of stop codons (TAA, TAG, and T). Among these, ATA serves as the initiation codon for three protein-coding genes (*atp6*, *nad6* and *cox2*). The ATC codon functions as the initiation codon for two protein-coding genes (*cox1* and *nad3*), while the ATT codon is employed as the initiation codon for two genes (*atp8* and *nad2*). The ATG codon is employed as the initiation codon for six protein-coding genes (*cox3*, *cytb*, *nad1*, *nad4*, *nad4L*, and *nad5*). Three types of stop codons are present (TAA, TAG, and T). Among these, TAA serves as the termination codon for six genes (*atp6*, *atp8*, *cytb*, *nad1*, *nad3*, and *nad6*), TAG is used by two genes (*nad2* and *nad4L*), and T is employed as the stop codon in five genes (*cox1*, *cox2*, *cox3*, *nad4*, and *nad5*). Collectively, ATG and TAA represent the most frequently utilized initiation and termination codons, respectively.

**Fig. 6:**
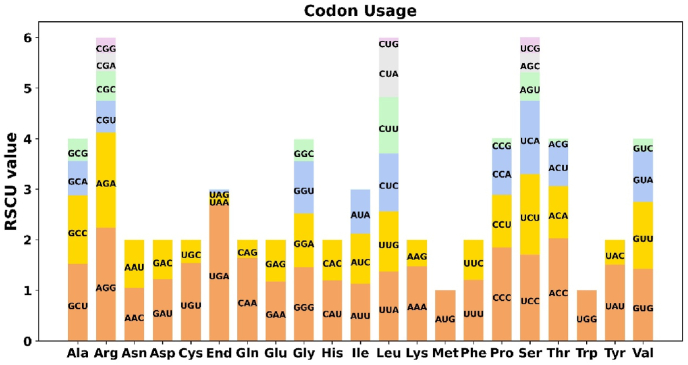
Codon usage bias in the mitochondrial genome.

Codon usage bias was analyzed for the 13 unique PCGs of *A. moniliformis*, and the codon usage patterns of individual amino acids are summarized in Fig. 6. Codons with a relative synonymous codon usage (RSCU) value greater than 1 are considered to be preferentially utilized for encoding amino acids. As shown in Fig. 6, except for the start codon AUG and the tryptophan codon UGG, both of which have an RSCU value of 1, the mitochondrial PCGs exhibit codon usage bias. For example, the stop codon UGA shows a strong usage preference, with the highest RSCU value (2.7) among the mitochondrial PCGs, followed by arginine (Arg), which preferentially utilizes AGG with an RSCU value of 2.24 (Table 3).

**Table 3:** Relative synonymous codon usage (RSCU) of individual amino acids in the mitochondrial genome of *Armillifer moniliformis*.

Amino	Codon 1	Codon 2	Codon 3	Codon 4	Codon 5	Codon 6
	RSCU	RSCU	RSCU	RSCU	RSCU	RSCU
Ala	GCU 1.52	GCC 1.36	GCA 0.68	GCG 0.44		
Arg	AGG 2.24	AGA 1.88	CGU 0.63	CGC 0.58	CGA 0.36	CGG 0.31
Asn	AAC 1.05	AAU 0.95				
Asp	GAU 1.22	GAC 0.78				
Cys	UGU 1.54	UGC 0.46				
Gln	CAA 1.64	CAG 0.36				
Glu	GAA 1.18	GAG 0.82				
Gly	GGG 1.46	GGA 1.06	GGU 1.03	GGC 0.44		
His	CAU 1.20	CAC 0.80				
Ile	AUU 1.13	AUC 0.99	AUA 0.88			
Leu	UUA 1.37	UUG 1.19	CUC 1.15	CUU 1.11	CUA 1.06	CUG 0.12
Lys	AAA 1.48	AAG 0.52				
Met	AUG 1.00					
Phe	UUU 1.21	UUC 0.79				
Pro	CCC 1.85	CCU 1.04	CCA 0.95	CCG 0.17		
Ser	UCC 1.71	UCU 1.59	UCA 1.45	AGU 0.56	AGC 0.38	UCG 0.32
TER	UGA 2.70	UAA 0.22	UAG 0.08			
Thr	ACC 2.03	ACA 1.03	ACU 0.86	ACG 0.08		
Trp	UGG 1.00					
Tyr	UAU 1.51	UAC 0.49				
Val	GUG 1.43	GUU 1.32	GUA 1.00	GUC 0.25		

### 3.7 rRNA and tRNA genes

The parasites had two rRNAs, including *rrn*12 of 657 bp and rrn16 of 1129 bp. The highest sequence identity of rrn12 of the parasites was 38.09 % with *Linguatula serrata* compared to other species in the *Linguatula* family, and rrn16 the highest sequence identity of 40.32 % with *A. grandis* (Table 4). The length of the 22 tRNAs ranged from 53 bp (trnC) to 66 bp (trnQ). The total length of the 22 tRNAs of the parasites was 1266 bp with an A + T content of 70 %; consequently, most codons were composed of A + T bases relative to G + C bases (Additional file 1: Fig. S1).

**Table 4:** Comparisons of nucleotide identity of PCGs, rRNA and NCRs of the mt genome of *Armillifer moniliformis* with the mt genomes of other Ichthyostraca species.

Region	Gene/region		Length of gene regions (bp)/nucleotide identity (%)					
		*Armillifer* sp.	*Armillifer*					*Linguatula*
			Armillifer armillatus		Armillifer agkistrodontis	Armillifer grandis	Armillifer moniliformis	*Linguatula serrata*
		Length(bp)	AY456186.1	MT271602.1	KX686568.1	KY914472.1	PV138266.1	MG951756.1
PCGs	*atp6*	651	654/75.27	654/75.12	654/71.12	654/74.19	651/99.69	657/58.49
	*atp8*	111	111/81.08	111/81.08	111/77.48	111/79.28	111/99.10	150/56.76
	*cox1*	1534	1525/82.95	1525/82.75	1525/81.31	1525/80.72	1525/99.67	1528/73.10
	*cox2*	664	664/83.43	664/83.28	664/86.60	664/82.83	664/99.85	667/70.39
	*cox3*	781	781/82.20	781/82.20	781/79.39	781/80.54	781/99.74	781/65.51
	*cytb*	1110	1110/82.56	1110/82.11	1110/78.86	1107/79.20	1110/99.73	1086/66.32
	*nad1*	909	918/83.39	918/83.39	905/80.86	918/84.6	909/99.78	915/31.92
	*nad2*	963	963/79.02	963/79.02	961/78.77	960/80.21	963/99.90	969/57.50
	*nad3*	345	345/76.81	345/76.52	343/80.47	343/75.80	345/99.42	349/64.72
	*nad4*	1264	1261/80.81	1261/80.33	1261/78.59	1261/79.54	1261/99.6	1262/62.18
	*nad4L*	270	270/86.30	270/84.14	270/82.96	270/85.19	270/99.63	264/68.18
	*nad5*	1603	1606/78.67	1606/78.42	1606/76.79	1606/78.79	1063/99.81	1555/63.52
	*nad6*	438	444/80.59	444/79.91	444/77.85	453/79.22	444/100	441/56.48
rRNA	*rrn12*	657	658/87.18	657/87.02	659/84.12	657/87.77	657/100	654/69.5
	*rrn16*	1129	1129/82.76	1129/82.49	1144/82.47	1146/37.88	1144/100	1128/38.17
CmtG		16516	16747	16705	16521	16073	16367	15328
TI(%)			82.8	82.4	81.3	82.4	99.79	73.99

CmtG, Complete mitochondrial genome; TI, total identity, null, no identity.

### 3.8 Analysis of repetitive sequences

**Fig. 7:**
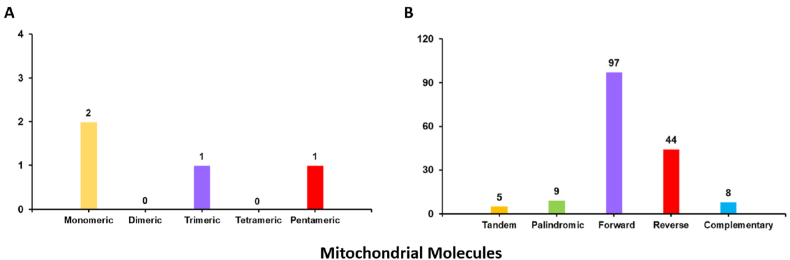
Histogram of dispersed repeats and simple sequence repeat (SSR) analysis. A. The x-axis represents SSR types, and the y-axis indicates the number of repeat motifs. Yellow bars correspond to mononucleotide SSRs, purple bars to trinucleotide SSRs, and red bars to pentanucleotide SSRs. No dinucleotide, tetranucleotide, or hexanucleotide SSRs were detected in this mitochondrial genome. B. Dispersed repeats. The x-axis represents the types of repeat sequences, and the y-axis indicates the number of repeat motifs. Yellow bars correspond to tandem repeats, green bars to palindromic repeats, purple bars to forward repeats, red bars to reverse repeats, and blue bars to complementary repeats.

The analysis of repetitive sequences in the mitochondrial genome of *A. moniliformis* is presented in Fig. 7. A total of four simple sequence repeats were identified, with mononucleotide SSRs accounting for 50 % of the total. Mononucleotide SSRs composed of adenine accounted for 100 % of the two mononucleotide SSRs identified. Dispersed repeats in the mitochondrial genome of *A. moniliformis*. Were analyzed, revealing a total of 158 repeat pairs with lengths ≥20 bp. Among these, 9 were palindromic repeats, 97 were forward repeats, 44 were reverse repeats, and 8 were complementary repeats. The longest palindromic, forward, reverse, and complementary repeats were 23bp, 1127bp, 27 bp, and 21 bp, respectively.

### 3.9 Comparative analysis of parasite mitochondrial genomes and the mitochondrial genome of Armillifer moniliformis

**Fig. 8:**
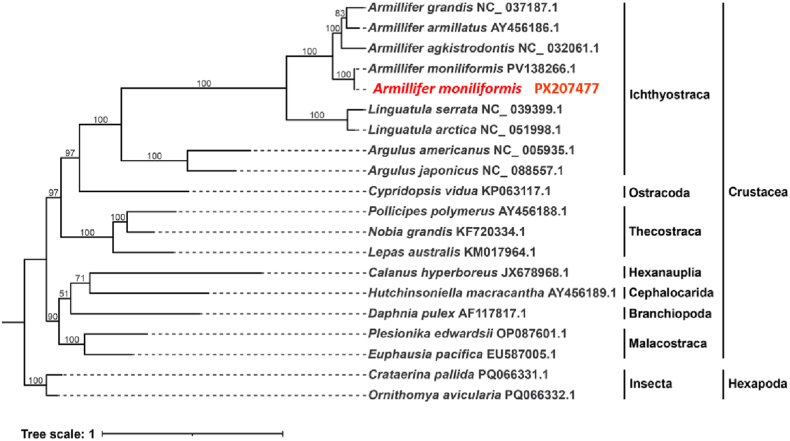
Phylogenetic tree based on the concatenated analysis of 13 mitochondrial protein-coding genes (PCGs) Phylogenetic tree of 13 PCGs sequences from the mt genomes in the families of *Insecta* and *Armillifer*. This tree is reconstructed based on the ML method. The numbers at the nodal points indicate the statistical values of the phylogenetic tree. Nodal points indicate bootstrap values. The scale bar represents the number of nucleotide substitutions per site.

A phylogenetic tree was constructed for 20 arthropod species based on the DNA sequences of 13 mitochondrial PCGs, as shown in Fig. 8. The 13 protein-coding genes included *atp6, atp8, nad1, nad2, nad3, nad4, nad4L, nad5, nad6, cox1, cox2, cox3,* and *cytb*. Two mitochondrial genomes from the subphylum Hexapoda were used as outgroups, and details of the best-fit model for tree construction are provided in Supplementary Table S4. This parasite belongs to the phylum Arthropoda, subphylum Crustacea, class Ichthyostraca, and clusters with species of the genus *Armillifer*.

This phylogenetic tree shows that sequences of *Armillifer* and *Linguatula* exhibit a close phylogenetic relationship, forming a clade with 100 % bootstrap support, and both belong to the subclass Pentastomida. This Pentastomida clade shares an evolutionary lineage with *Argulus* spp. In the subclass Branchiura and *Cypridopsis vidua* in the subclass Ostracoda, together forming the superclass Oligostraca. Results based on the concatenation approach indicate that the Pancrustacea clade, consisting of Crustacea and Hexapoda, is a monophyletic group with high bootstrap support for each branch, suggesting that they originated from a single common ancestor.

## 4 Discussion

Currently, the genus *Armillifer* comprises eight species, all of which can release eggs through the excreta and saliva of their definitive hosts. These eggs can be detected by microscopic examination of the secretions. Furthermore, when the definitive host preys on the intermediate host, the parasite larvae develop within the definitive host. Meanwhile, when the feces or saliva of the definitive host contaminates food or water sources, the intermediate host also becomes infected, thereby completing the bidirectional infection process between hosts in the parasite transmission cycle. However, the diagnosis of infection in intermediate hosts currently relies primarily on imaging techniques, exploratory laparotomy, and post-mortem examination of deceased animals(Tappe and Büttner, 2009). Therefore, there is an urgent need to develop precise molecular approaches for the accurate detection of parasite infections in intermediate hosts.

The oral hooks on the head of *A*. *armillatus* and *A*. *agkistrodontis* are arranged in a single row, with no distinction between upper and lower oral hooks. In contrast to *A*. *agkistrodontis*, *A*. *moniliformis* has a greater number of ventral annuli. However, the *A*. *moniliformis* in this study has a flattened head, with 4 oral hooks located below, symmetrically arranged on both sides of the oral aperture. The middle ventral annulus of *A*. *moniliformis* is the widest, and its surface is covered with irregularly arranged serrated protrusions a phenomenon not reported in other studies on *A*. *moniliformis*. The morphological differences in the head mouthparts we observed are likely attributed to sexual dimorphism. Specifically, similar morphological variations have been documented in *A. agkistrodontis* in prior studies, where such differences were confirmed to be associated with gender. Based on this existing evidence, we infer that the morphological variations of *A. agkistrodontis* observed in our study may also result from sexual differences(Rajapaksha et al., 2020; Chen et al., 2010; Vibulchan et al., 2024).

The complete mitochondrial genome of *A. moniliformis* is 16516 bp in length, with a non-coding region of 2765 bp, which is slightly shorter than that of *A*. *agkistrodontis* (16521 bp; non-coding region 2769 bp) and *A*. *armillatus* (16705 bp; non-coding region 2951 bp)(Li et al., 2016; Lemarcis et al., 2020). It is longer than the mitochondrial genome of *A*. *grandis* (16073 bp; non-coding region 2317 bp)(Grau et al., 2017). The mitochondrial genome of *A*. *moniliformis* is 16367 bp in length, with a non-coding region of 2616 bp(Pataradool et al., 2025). The mitochondrial genome of *Linguatula serrata* is 15328 bp in length(Naude et al., 2018). The gene order of the mitochondrial genome of *A. moniliformis* is similar to that of *A*. *moniliformis*. ATG and TAA are the most frequently used start and stop codons in *A. moniliformis*, respectively, which is consistent with the patterns observed in *A*. *moniliformis* and *A*. *agkistrodontis*(Li et al., 2016; Pataradool et al., 2025). However, this difference requires further verification by means of transcripts obtained through transcriptome sequencing. Nevertheless, the *cox*1 gene in *A*. *grandis*, *A*. *armillatus*, *A*. *agkistrodontis*, and *A*. *moniliformis* uses CTG as the start codon, whereas in *Armillifer* sp., ATC serves as the start codon. This difference may result from a combination of factors, including natural selection and GC content(Yang et al., 2014).

Using the Tandem Repeats Finder software, we identified a total of four tandem repeats in the mitochondrial genome of *A. moniliformis*. Each fragment exhibited distinct period sizes and A + T content. Among these, the fourth repeat fragment was the longest, measuring 1658 bp, and consisted of five tandem repeats. It also had the highest A + T content, reaching 71.0 %. Similar high A + T bias was also observed in the longest NCR repeat fragment of each species, namely *A*. *moniliformis*, *A*. *armillatus*, *A*. *grandis*, and *A*. *agkistrodontis*. This suggests that the tandem repeat sequence may facilitate mitochondrial DNA replication in species of this genus by serving as binding sites for replication complexes or forming the secondary structures required for the function of the origin of replication.

Molecular marker technology has been used to clarify the phylogenetic taxonomic status of Pentastomida within Crustacea, providing a basis for the analysis of the phylogenetic relationships among taxa(Lavrov et al., 2004). Among these, the 18S rRNA gene, due to its high stability, has long been regarded as a core molecular marker for resolving the molecular phylogenetic relationships among parasite genera, and has been successfully applied in studies related to the genus *Armillifer*. For example, this gene has been successfully applied to resolving the molecular phylogenetic relationships of *A*. *agkistrodontis* within the genus *Armillifer* (Chen et al., 2010).

For this study, we selected 19 complete mitochondrial genomes from GenBank for phylogenetic analysis. As shown in this phylogenetic tree, the target gene *A*. *moniliformis* PX207477 forms a clade with *A*. *moniliformis*, *A. agkistrodontis*, *A*. *armillatus*, and *A*. *grandis* with 100 % bootstrap support, all belonging to the genus *Armillifer*. This genus and the genus *Linguatula* together form a clade of the subclass Pentastomida. This Pentastomida clade shares an evolutionary lineage with *Argulus americanus* and *Argulus japonicus* of the subclass Branchiura, as well as *Cypridopsis vidua* of the subclass Ostracoda, collectively forming the superclass Oligostraca. Based on the topology, the Pancrustacea clade, consisting of Crustacea and Hexapoda, is a monophyletic group with high bootstrap support for each branch, suggesting that they originated from a single common ancestor.

Previous studies have reported that the definitive hosts of *Armillifer* sp. Parasites are primarily reptiles, particularly snakes, including *Bitis* vipers, *Bitis nasicornis*, and *Sanzinia madagascariensis*(Hardi et al., 2017; Yan et al., 2021; Grau et al., 2017). Intermediate hosts include humans and various mammals, such as long-tailed macaques (*Macaca fascicularis*), palm civets (*Paradoxurus montanus*), pottos (*Perodicticus potto*), leopards (*Panthera pardus*), and hyenas (*Hyaena*), all of which can become infected(Rajapaksha et al., 2020; Junker and de Klerk-Lorist, 2020; Li et al., 2024; Li et al., 2016; Lemarcis et al., 2020; Pataradool et al., 2025). To date, there have been no reported cases of *Armillifer* sp. Infection in pangolins. Cases of *Armillifer* sp. Infection are predominantly reported in economically underdeveloped regions, such as parts of Gambia, Congo, Thailand, and Malaysia. In these areas, infections primarily result from the consumption of undercooked snake meat, snake blood, or other snake tissues, or exposure to contaminated water sources(Pataradool et al., 2025; Tappe et al., 2011; Ramachandran et al., 2023). *Armillifer* sp. Infection can cause a range of clinical manifestations in hosts, including abdominal pain, intestinal obstruction, necrotizing enteritis, blindness, hepatic encephalopathy, hydrosalpinx, and neurological symptoms. In severe cases, the infection may result in host death(Lavarde and Fornes, 1999; Ramachandran et al., 2023; Ioannou and Vamvoukaki, 2019; Tappe et al., 2014). Currently, the treatment of *Armillifer* sp. Infections can be divided into two approaches: surgical removal of affected tissues may be considered in severe cases, whereas mild infections can be managed with antiparasitic drugs, such as oral albendazole(Ioannou and Vamvoukaki, 2019). In intermediate hosts, *Armillifer* sp. Can form cysts in various tissues, with the vast majority primarily localized in abdominal organs, and only rarely in the thoracic cavity(Chen et al., 2010; Tappe and Büttner, 2009). Histopathological examination of infected tissues revealed mild hemorrhage, leukocyte infiltration, and slight hepatocellular degeneration in the liver. *Armillifer* sp. Parasites were observed within thin-walled cysts, accompanied by a small number of inflammatory cells, and granuloma formation occurred at the sites of parasitism(Rajapaksha et al., 2020). Similar pathological changes were observed in organ tissue sections of pangolin-derived *A. moniliformis*.

In summary, *Armillifer* sp. Can parasitize a variety of intermediate and definitive hosts; however, no systematic studies have been conducted on *A. moniliformis* infection in Malayan pangolins. This study systematically characterizes pangolin-derived *A. moniliformis* in terms of morphology, histopathology, and mitochondrial genomics, providing insights into its pathological features and molecular evolution. These findings provide a foundational basis for diagnosing, controlling, and further studying *A. moniliformis* infections in pangolins and related parasitic diseases.

## 5 Conclusion

In this study, the mitochondrial genome of *A. moniliformis* parasites from a deceased Malayan pangolin was sequenced using Illumina and HiFi technologies. This work fills the current knowledge gap regarding *A. moniliformis* infections in pangolins and provides a systematic description of the larval morphology. It enriches the foundational database of pangolin parasitic diseases and provides critical genomic information for *A. moniliformis* Additionally, a phylogenetic tree was constructed based on protein-coding gene sequences, elucidating the evolutionary relationships and taxonomic position of this parasite relative to closely related species.

## CRediT authorship contribution statement

**Xianghe Wang:** Writing – review & editing, Writing – original draft, Methodology, Data curation, Conceptualization. **Sitong Chen:** Project administration, Formal analysis, Data curation. **Fuyu An:** Software, Project administration. **Xinyu Liu:** Validation, Supervision. **Hongmei Yan:** Visualization, Validation, Data curation. **Zhenquan Zhang:** Visualization, Investigation. **Zi-Guo Yuan:** Writing – review & editing, Conceptualization. **Yan Hua:** Writing – review & editing, Funding acquisition, Conceptualization.

## Data availability statement

The data for this study have been published on NCBI (http://ncbi.nlm.nih.gov/) and can be found by serial number PX242633 and PX207477. The data supporting this study's findings are available upon reasonable request.

## Ethics approval and consent to participate

All the animal experiment and sample collection procedures were approved by the Guangdong Academy of Forestry (00202671 − February 4, 5022). Administration and received support and permission from the Guangdong Provincial Wildlife Rescue Monitoring Center (200932 − 11/2/2022). Trained veterinarians obtained all the samples, following standard routine procedures. All of our methods were complied with the statement of the ARRIVE guidelines report. All methods were carried out in accordance with relevant guidelines and regulations. The present study was obtained from legal authorization of Guangdong Wildlife Rescue Monitoring Center.

## Publisher's note

All claims expressed in this article are solely those of the authors and do not necessarily represent those of their affiliated organizations, or those of the publisher, the editors, and the reviewers. Any product that may be evaluated in this article, or claim its manufacturer may make, is not guaranteed or endorsed by the publisher.

## Funding

This study was funded by the National Key Program of Research and Development, 10.13039/100007225Ministry of Science and Technology (No. 2025YFC3507700).

## Conflict of interest

The authors declare that the research was conducted in the absence of any commercial or financial relationships that could be construed as a potential conflict of interest.

## Abbreviations:

***atp 6 and atp 8***: Genes coding ATPase subunits 6 and 8, respectively
***cox 1–3***: Cytochrome *c* oxidase subunits 1-3
***cytb***: Cytochrome *b*
***MtDNA***: Mitochondrial DNA
***nad 1-6 and nad 4L*:**: NADH dehydrogenase subunits 1–6 and 4L, respectively
***PCG***: Protein-coding gene
***rRNA***: Ribosomal ribonucleic acid
***tRNA***: Transfer RNA
***SEM***: Scanning Electron Microscopy
***CCS***: Circular Consensus Sequencing
***RSCU***: Relative Synonymous Codon Usage
***ML:***: Maximum Likelihood
