# Supplementary material for: Complete mitochondrial genome pathological characteristics and scanning electron microscopic observations of Armillifer moniliformis isolated from Manis javanica
Source: Int J Parasitol Parasites Wildl. 2026 Jan 3;29:101183. doi: 10.1016/j.ijppaw.2025.101183 (PMC12856591; doi:10.1016/j.ijppaw.2025.101183)
Supplement: Multimedia component 1 [file mmc1.docx]

**Supplementary Tables and Figures**


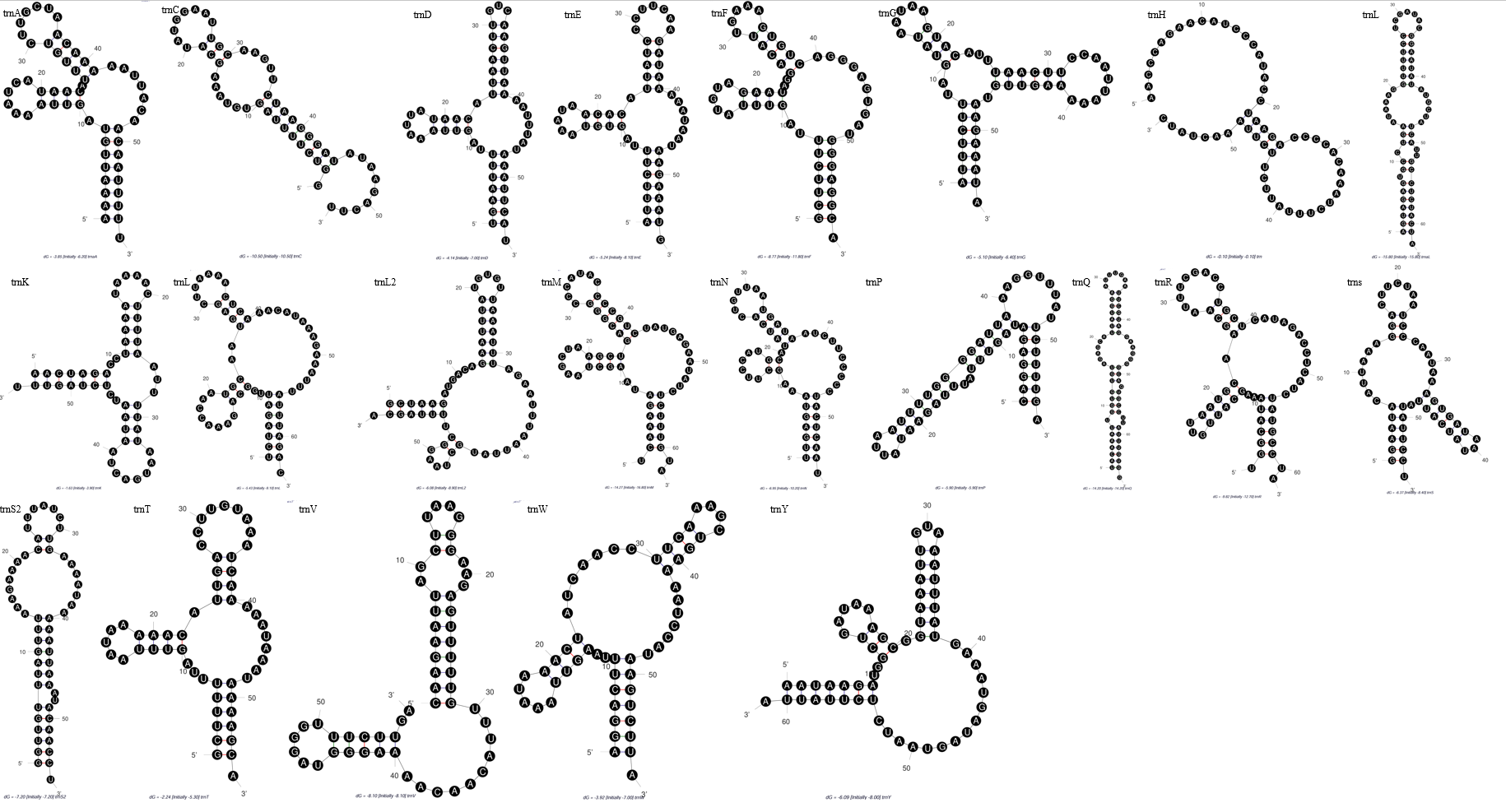


**Supplementary** **Figure S1.** Predicted secondary structure of tRNA genes in the *Armillifer moniliformis* mt genome.

**Supplementary Table S1.** Retrieved 18S rRNAs in species of Ichthyostraca from GenBank.

| **Family** | **Species** | **Size (bp)** | | **Accession number** |
| --- | --- | --- | --- | --- |
| Armilliferidae | *Armillifer moniliformis* | | 1836 | HM048870.1 |
|  | *Armillifer moniliformis* | | 1842 | PV124096.1 |
|  | *Armillifer moniliformis* | | 1836 | ON982766.1 |
|  | *Armillifer agkistrodontis* | | 1835 | KX686569.1 |
|  | *Armillifer agkistrodontis* | | 1835 | FJ607339.1 |
|  | *Armillifer sp.* | | 1835 | LC695012.1 |
|  | ***Armillifer moniliformis*** | | **1834** | **PX242633.1** |
| Linguatulidae | *Linguatula serrata* | | 1830 | KP100453.2 |
|  | *Linguatula serrata* | | 1830 | JX088397.2 |
|  | *Linguatula arctica* | | 1830 | KF029442.1 |
|  | *Linguatula arctica* | | 1830 | KF029441.1 |
| Raillietiellidae | *Raillietiella orientalis* | | 1776 | KC904945.1 |

**Supplementary Table S2**. The mt genome from order of Crustacea and Hexapoda.

| **Family** | **Species** | **Size (bp)** | **Accession number** |
| --- | --- | --- | --- |
| Ichthyostraca | *Armillifer grandis* | 16073 | NC037187.1 |
|  | *Armillifer armillatus* | 16747 | AY456186.1 |
|  | *Armillifer agkistrodontis* | 16521 | NC032061.1 |
|  | *Armillifer moniliformis* | 16367 | PV138266.1 |
|  | *Linguatula serrata* | 15328 | NC039399.1 |
|  | *Linguatula arctica* | 14789 | NC051998.1 |
|  | *Argulus americanus* | 15102 | NC005935.1 |
|  | *Argulus japonicus* | 15062 | NC088557.1 |
|  | ***Armillifer moniliformis*** | **16516** | **PX207477** |
| Ostracooda | *Cypridopsis vidua* | 16783 | KP063117.1 |
| Thecostraca | *Pollicipes polymerus* | 15634 | AY456188.1 |
|  | *Nobia grandis* | 15032 | KF720334.1 |
|  | *Lepas australis* | 15502 | KM017964.1 |
| Hexanauplia | *Calanus hyperboreus* | 17910 | JX678968.1 |
| Cephalocarida | *Hutchinsoniella macracantha* | 16491 | AY456189.1 |
| Branchiopoda | *Daphnia pulex* | 15333 | AY117817.1 |
| Malacostraca | *Plesionika edwardsii* | 15956 | OP087601.1 |
|  | *Euphausia pacifica* | 16898 | EU587005.1 |
| Insecta | *Crataerina pallida* | 21552 | PQ066331.1 |
|  | *Ornithomya avicularia* | 18431 | PQ066332.1 |

| **Supplementary Table S3.** Nucleotide composition (%) of protein coding genes (PCGs), entire mt genome, and skew value of *Armillifer moniliformis* | | | | | | | | |
| --- | --- | --- | --- | --- | --- | --- | --- | --- |
| **Gene** | **A** | **T** | **C** | **G** | **A+T (%)** | **G+C (%)** | **AT skew** | **GC skew** |
| *atp6* | 26 | 35 | 35 | 4 | 61 | 39 | -0.12 | -0.78 |
| *atp8* | 40 | 29 | 28 | 3 | 69 | 31 | 0.20 | -0.77 |
| *cox1* | 25 | 35 | 29 | 11 | 60 | 40 | -0.14 | -0.43 |
| *cox2* | 31 | 32 | 29 | 8 | 63 | 37 | 0.00 | -0.56 |
| *cox*3 | 25 | 36 | 32 | 7 | 61 | 39 | -0.14 | -0.61 |
| *cytb* | 29 | 30 | 35 | 6 | 59 | 41 | 0.00 | -0.69 |
| *nad*1 | 39 | 18 | 37 | 6 | 57 | 43 | -0.01 | -0.76 |
| *nad*2 | 29 | 39 | 27 | 5 | 68 | 32 | -0.13 | -0.65 |
| *nad*3 | 26 | 37 | 31 | 6 | 63 | 37 | -0.13 | -0.64 |
| *nad4* | 18 | 42 | 5 | 35 | 60 | 40 | 0.36 | 0.75 |
| *nad*4L | 19 | 41 | 1 | 39 | 60 | 40 | 0.67 | 0.91 |
| *nad*5 | 21 | 41 | 5 | 33 | 62 | 38 | -0.29 | 0.73 |
| *nad*6 | 29 | 30 | 37 | 4 | 59 | 41 | 0.02 | -0.80 |
| *rrn*12 | 29 | 40 | 5 | 26 | 69 | 31 | -0.11 | 0.64 |
| *rrn*16 | 28 | 40 | 6 | 26 | 68 | 32 | -0.15 | 0.58 |

Cmtg: Complete mitochondrial genome, E: entire (PCG, tRNA, rRNA, NCR)

**Supplementary Table S4.** List of the best-performing substitution models for each partition in phylogenetic analysis using the full concatenation approach

| Gene/partition | Substitution model |
| --- | --- |
| *atp6* | TPM3u+F+I+G4 |
| *atp8* | TVM+F+I+G4 |
| *nad1* | K3Pu+F+I+G4 |
| *nad2* | TVM+F+I+G4 |
| *nad3* | HKY+F+I+G4 |
| *nad4* | TVM+F+I+G4 |
| *nad4L* | K3Pu+F+G4 |
| *nad5* | TVM+F+I+G4 |
| *nad6* | TN+F+I+G4 |
| *cox1* | GTR+F+I+G4 |
| *cox2* | TIM+F+I+G4 |
| *cox3* | TIM+F+I+G4 |
| *cytb* | K3Pu+F+I+G4 |

GTR：General time reversible model with unequal rates and unequal base frequency

HKY：Unequal transition/transversion rates and unequal base frequency

TN：Like HKY but unequal purine/pyrimidine rates

K3Pu：Three substitution types model and unequal base frequency

TIM：Transition model, AC=GT, AT=CG and unequal base frequency

TPM3u：AC=CG, AG=CT, AT=GT and unequal base frequency

TVM：Transversion model

+F：Empirical base frequencies

+G：Discrete Gamma model

+I：Invariable sites

| **Supplementary Table S5. Statistical table of repeat sequences in the mitochondrial genome of *Armillifer moniliformis*.** | | | | | | | | | | | | |
| --- | --- | --- | --- | --- | --- | --- | --- | --- | --- | --- | --- | --- |
| Indices | Period | Copy | Consensus | Percent | Percent | Score | | A | C | G | T | Entropy |
|  | Size | Number | Size | Matches | Indels |  |  |  |  |  |  | (0-2) |
| [13478--13502](https://tandem.bu.edu/trf/output/tmp3ljkbo3a.2.7.7.80.10.50.500.1.txt.html#13478--13502,12,2.1,12,1) | 12 | 2 | 12 | 100 | 0 | 50 | 40.00 | | 28.00 | 0.00 | 32.00 | 1.57 |
| [13750--14443](https://tandem.bu.edu/trf/output/tmp3ljkbo3a.2.7.7.80.10.50.500.1.txt.html#13750--14443,44,15.8,44,2) | 44 | 16 | 44 | 100 | 0 | 1388 | 27.00 | | 50.00 | 13.00 | 9.00 | 1.72 |
| [14523--14551](https://tandem.bu.edu/trf/output/tmp3ljkbo3a.2.7.7.80.10.50.500.1.txt.html#14523--14551,13,2.2,13,5) | 13 | 2 | 13 | 100 | 0 | 58 | 13.00 | | 58.00 | 0.00 | 27.00 | 1.36 |
| [14625--16283](https://tandem.bu.edu/trf/output/tmp3ljkbo3a.2.7.7.80.10.50.500.1.txt.html#14625--16283,332,5.0,332,6) | 332 | 5 | 332 | 99 | 0 | 3300 | 36.00 | | 17.00 | 10.00 | 35.00 | 1.84 |


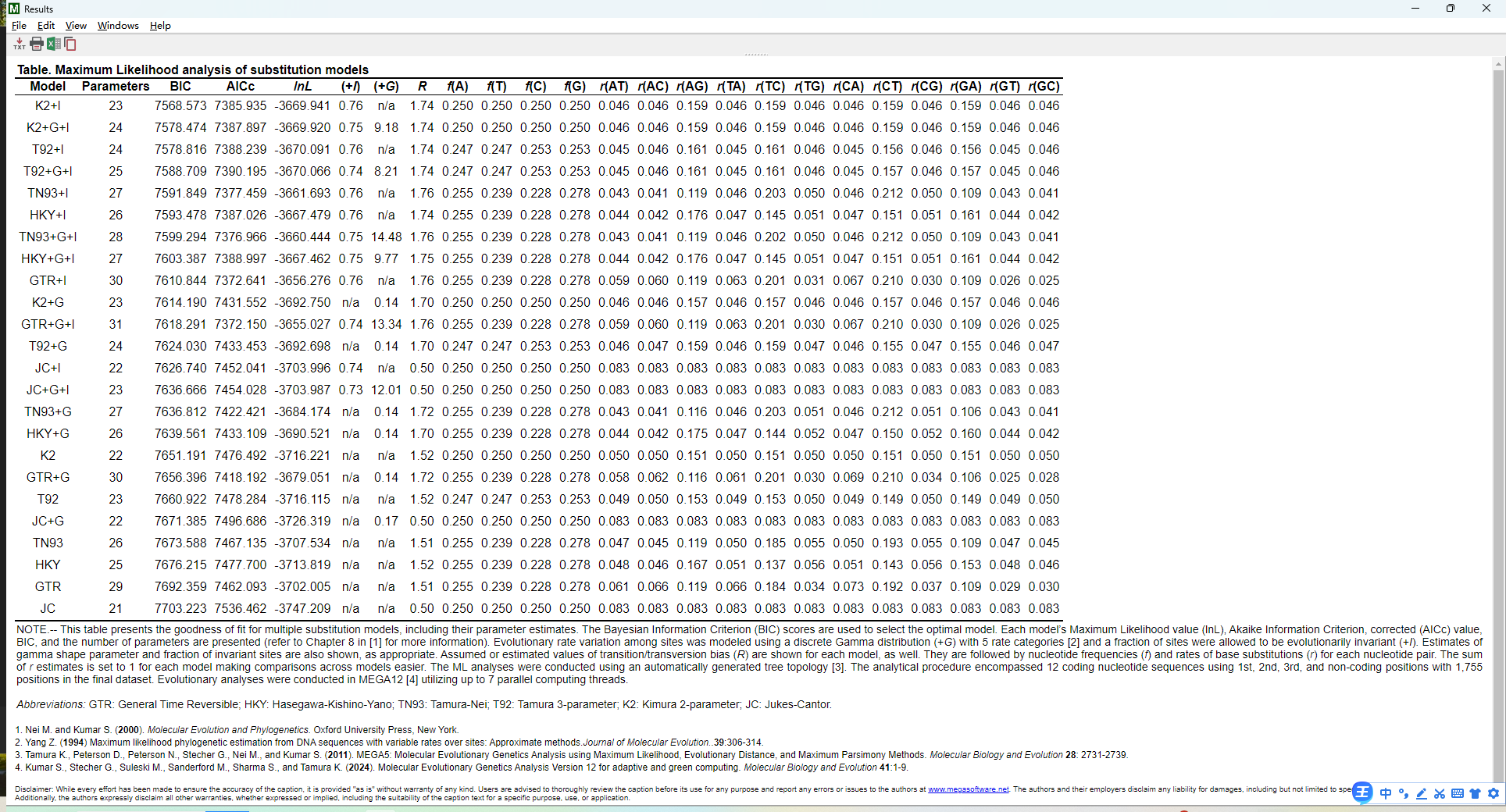


**Supplementary Figure S2. This table details the model parameters and justification, providing transparency on the methodological choices.**
